# Demonstration and application of diffusive and ballistic wave propagation for drone-to-ground and drone-to-drone wireless communications

**DOI:** 10.1038/s41598-020-71733-0

**Published:** 2020-09-08

**Authors:** Peter J. Burke

**Affiliations:** EECS, UC Irvine, Irvine, CA 92617 USA

**Keywords:** Aerospace engineering, Electrical and electronic engineering

## Abstract

In order to determine how an electromagnetic wave propagates from a base station to a cell phone or a wirelessly connected device, we use a novel Unmanned Aerial Vehicle (UAV) mapping technology to map the cellular network coverage at various altitudes in various terrains (flat, hilly, mountainous). For the flat terrains, the waves are shown to propagate ballistically: They have an altitude independent path loss consistent with minimal scatter in the propagation from transmitter to (aerial) receiver. In mountainous terrain, the waves are shown to propagate in the diffuse regime, and demonstrate a 10 dB increase in received signal intensity per 100′ of altitude gain, up to 400′. In the intermediate case, evidence of coherent wave interference is clearly observed in altitude independent interference patterns. These general observations can be used to build a physical or empirical model for drone-to-ground and drone-to-drone propagation, for which existing models are shown to fail. While important for building physical models of wave propagation in wireless networks, this method can be used more generally to determine the magnitude and phase of an electromagnetic wave at every point in space, as well as usher in the era of drone-to-ground and drone-to-drone communications.

## Introduction

How does an electromagnetic wave propagate from a base station to a cell phone or a wirelessly connected device (such as in the internet of things)? Ever since Marconi invented wireless communications over a century ago, the propagation of waves from sender to receiver has been studied and exploited in ways that have forever changed the way every human on the planet interacts. Of all the frequencies in the electromagnetic spectrum, the microwave range has been the most exploited because of its bandwidth and propagation characteristics, which uniquely enable both line of sight long range propagation including as far as from satellites to earth as well as short range propagation such as Wi-Fi routers in buildings and homes where the waves bend, scatter, and diffract around walls and other obstacles to provide coverage even in the absence of a direct, clear path from sender to receiver. These two realms of wave propagation depend on the number of scattering centers between transmitter and receiver, just as the resistance of a metal depends on the number of scattering sites for electron waves. In both electron (quantum wave function) waves and electromagnetic (micro) waves, the two extremes can be treated as ballistic (minimal scattering between source/detector) and diffusive (large number of scattering events between source and detector).

In general, actually measuring the microwave propagation in three dimensions over large areas has been a challenge, leading the entire telecommunications industry to base most of the deployment of billions of dollars of wireless infrastructure around the world on models of scattering that are derived from surprisingly few measurements that were performed at the ground level only (i.e. in only 2 dimensions) almost half a century ago^1–3^.

Here we show measurements of signal propagation in 3 dimensions which demonstrate the transition from ballistic to diffusive wave propagation in an actual deployed wireless communication network (a 4G internet cellular wireless provider). (A preliminary version of this work was presented in a conference paper in^4^.) By utilizing an unmanned aerial vehicle to map the propagation in three dimensions, we demonstrate that the waves can propagate in the ballistic and diffusive regime, depending on the nature of the local terrain and environment. In the ballistic regime the wave intensity follows the behavior of classical electromagnetic waves in free space without scattering, i.e. it is independent of altitude from ground level to 150 m indicating minimal scatter between the ground based transmit station and the UAV, whereas in the diffusive case the wave pattern is complex, distributed, and depends on altitude in a terrain dependent way, consistent with the expectation that diffuse wave propagation is dominantly determined by the number and density of scattering sites. Interference phenomenon can be mapped with exquisite detail, illustrating the wave nature of the propagation depends not only on the number and density of scattering sites, but the relative phases of the multiple paths from source to detector.

Elucidating the propagation characteristics in these two regimes is enabled by the UAV technology, the first new technology in over a century to map electromagnetic wave propagation in 3D, which was previously impossible with ground based measurements alone, and which was prohibitively expensive and unsafe at low altitudes with manned aircraft, especially in mountainous terrain.

This work demonstrates the potential to map experimentally (rather than model theoretically) the wireless coverage over every point on the entire Earth, something which wireless carriers have always dreamed of. Quantitative models generated with such measurement data could revolutionize the coverage mapping, predictions, and construction of wireless network infrastructure, and save billions of dollars while improving signal connection integrity, bandwidth, fidelity, and efficiency, and (potentially) enabling entire new fields of wireless communications techniques and systems based on precise, detailed, and intimate knowledge of signal channel propagation, including both the magnitude and the phase of the wave at every point in space.

**Figure 1:**
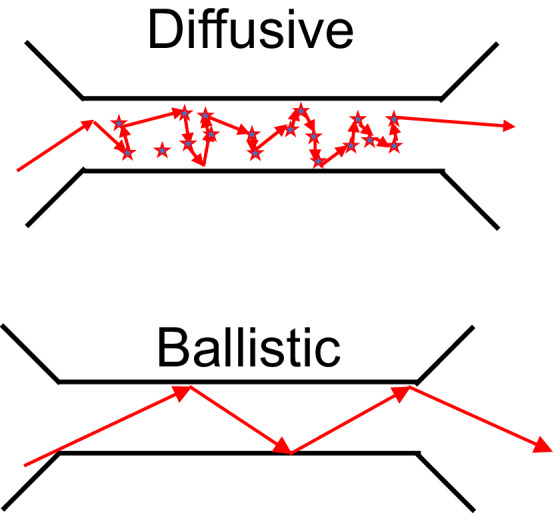
Ballistic vs. diffusive propagation of waves.

**Figure 2:**
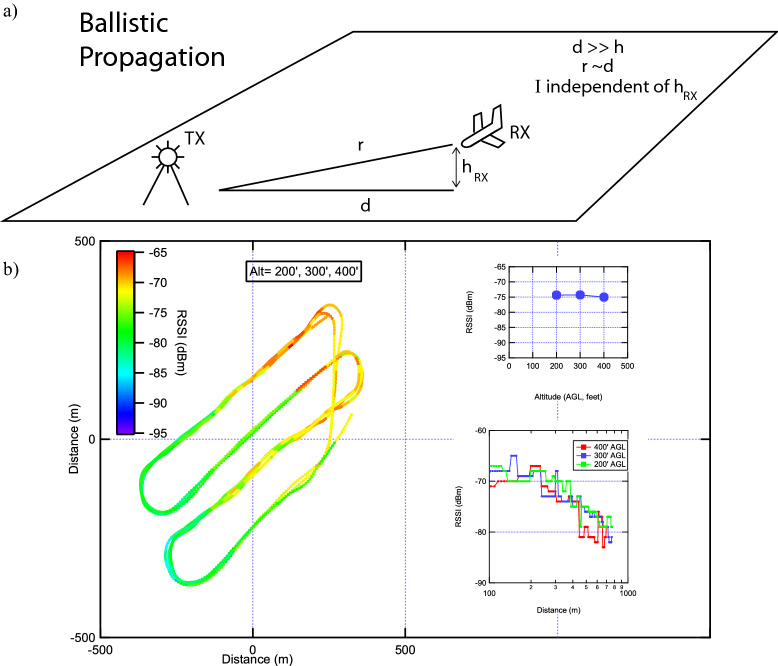
Top panel: Schematic of propagation from base station to airborne receiver (UAV). Bottom panel: UAV flight path color coded by RSSI at three different altitudes (100′, 200′, 300′). The data are almost identical when plotted separately at each altitude. Upper right inset: Average RSSI vs. altitude for the entire flight at each altitude, showing very little dependence on the altitude. Lower right inset: RSSI vs. distance from the northeast most flight point plotted along the northernmost flight leg at three different altitudes.

**Figure 3:**
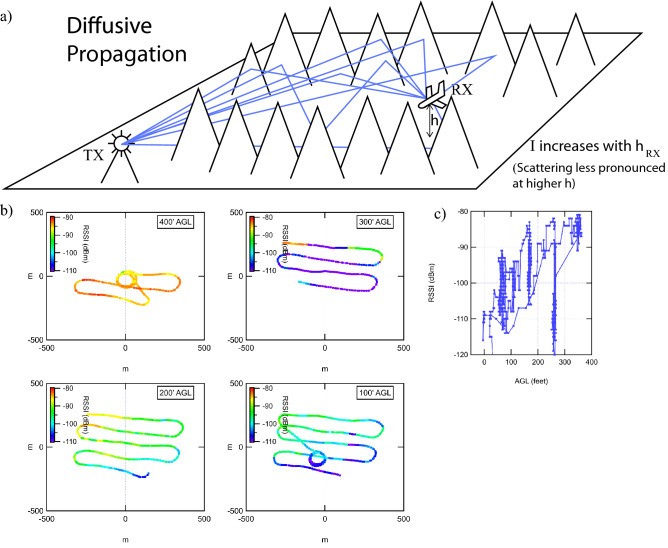
Top panel: Schematic of propagation from base station to airborne receiver (UAV). The blue lines are example rays the microwave may follow from base station the UAV. Bottom panel: UAV flight path color coded by RSSI at four different altitudes (100′, 200′, 300′, 400′). Upper Right panel: RSSI vs. altitude for the entire flight, showing   10 dB of signal gain per 100′ of altitude gain.

Figure 1 shows how the propagation of waves, whether it be electron waves or electromagnetic waves, can be divided into two regimes: Ballistic, where the wave propagates from point A to point B without scatting, and diffusive, where the wave propagates from point A to point B after many scattering events.

The scattering events can be off of static or dynamic scattering centers. The theory of scattering off of random media has been in the literature for some time^5^. However, in the realm of propagation of wireless signals in the GHz range, the measurement of the propagation has been severely limited due to the lack of a technology to map in three dimensions the wave propagation. Therefore, models have mostly been used to predict coverage areas, and billions of dollars of infrastructure are based on these theoretical models. We review these models^1–3,6–10^ and discussed how they relate in detail to our measurements in this manuscript, but briefly they are limited to 2D and our measurement technique enables truly 3D signal mapping.

The use of UAVs for this is a perfect use case, since a UAV has the following characteristics:

- Can move in arbitrary 3D positions
- Can explore regions that have no access via roads, etc.
- Can accurately measure position and field strengths
- Are economical, safe, and quiet compared to manned aircraft attempting the same mission.

Most UAVs are several kg or heavier, with flight time limited to under 30 min. We have recently developed a 1 hour flight time UAV with AUW < 300 g^11^. This platform is ideal for the 4G, 5G, and beyond signal mapping missions, since it is quiet, safe, economical, and has a long flight time. We use this platform to map wave intensity in several different terrain environments to study the wave propagation characteristics.

## Results

Three locations in Southern California were chosen mainly out of convenience, safety, and regulatory concerns. The flight path was pre-programmed on the computer and the UAV flew the waypoint missions autonomously, with the pilot in command able to take control at any point if needed (it wasn’t needed). All missions were flown with appropriate FAA and other permissions as needed, and flown according to FAA regulations, keeping the aircraft in line of sight for the entire flight. The missions were mostly designed out of curiosity and no a-priori hypothesis about signal strength was used in the mission planning stage.

The on board 4G modem provided an RSSI (received signal strength indicator) through a software interface that was recorded at each position in space in units of dBm (dB relative to 1 mW). (See “Methods”). Therefore, the RSSI is a direct measure of the signal strength.

### Flight A: ballistic wave propagation characteristics

Since the platform is a flying wing, we mapped the RSSI in a grid pattern at several different flight altitudes. The flight pattern is shown in Fig. 2b, with a color scale indicating the RSSI at that particular location. The same grid was flown at three different altitudes (200′, 300′, and 400′ AGL). The data show some clear trends: First, and most strikingly, the signal strength pattern is the same at all three altitudes. In fact, the three different flight paths overlap exactly in color, indicating that only the horizontal (xy) position determines the signal strength, and not the altitude.

In order to more closely examine the altitude dependence, we plot the average RSSI vs. altitude for three altitudes. The signal strength (i.e. RSSI) is clearly independent of altitude. This is not seen in scattering models (see discussion section), indicating that the characteristic of the propagation in this case is not dominated by scattering, but rather the direct line of sight from the base station to the UAV receiver.

The next major feature of this data is the overall increase in signal strength vs. position (in the northeast direction). This indicates the nearest base station is indeed to the northeast. In order to demonstrate that the dependence on distance to the base station is independent of altitude, we plot the RSSI vs. distance from the northeast most flight point in the inset for three different altitudes, demonstrating clearly an altitude-independent path loss.

The propagation is not entirely ballistic, though, because the power law decay would be given by the Friis transmission loss formula in the absence of any scattering, i.e.

1 $$\begin{aligned} P_R=P_T + D_{TX} + D_{RX} + 20 log(\lambda /4\pi d), \end{aligned}$$

where $P_T$ is the transmit power, $P_R$ the receive power, $D_{TX}$ the transmitter directivity, $D_{RX}$ the receiver directivity, d the distance to the transmitter, and $\lambda$ the wavelength. Although the power law decay of P vs. d cannot be determined because the precise distance to the transmitter is not precisely known, it can be determined based on estimates that the decay falls faster than 20 dB per decade as expected by the Friis formula. This indicates the wave propagation is not entirely ballistic, and will be discussed in more detail in the discussion section below.

### Flight B: diffusive wave propagation characteristics

Flight B was performed in mountainous terrain where the flight is surrounded by mountains in a valley. The map of the signal strength at four different altitudes (100′, 200′, 300′, and 400′ AGL) is shown in Fig. 3. In stark contrast to the first flight (flight A), the overall signal strength is very dependent on altitude. In Fig. 3c we plot the signal strength vs. altitude for the entire flight. From this plot we see that, for this flight, we observe an altitude dependence showing about 10 dB of increase in signal strength per 100′ gain in altitude. The flights took place in a canyon, and we interpret this to cause the scattering to become weaker at higher altitudes where the mountainous terrain thins out.

We also observe the signal strength to be stronger in the northwest quadrant vs. the southeast quadrant. This indicated the relative direction to the cell tower. Switching between different cell towers explains the sudden change in signal strength for the 300′ data.

To compare the two flights (A, B): In B, the signal strength is strongly dependent on altitude and weakly dependent on position. In A, the signal strength has almost no dependence on altitude and is very strongly dependent on position. This leads us to conclude that, in flight B, the signal propagation from base station to the UAV undergoes many scattering events (probably off the walls of the canyon in this case), i.e. the wave propagation is in the ballistic regime. Note that existing models^1–3,6–10^, which do model diffuse scattering in flat terrains but with building, trees, etc. do not model large mountains as we have measured here, and do not cover altitudes up to what we have measured here.

We want to note that, other than Qualcomm study (discussed below), these are the first comprehensive data sets of microwave signal propagation in the wireless regime at moderate altitude (100–400′). Therefore, the initial modeling and interpretation can only qualitatively capture the main features of ballistic vs. diffusive propagation of microwaves in the wireless range. It is our hope that these initial measurements and insights will form the basis for more comprehensive models of drone-to-ground and drone-to-drone signal propagation and communications.

**Figure 4:**
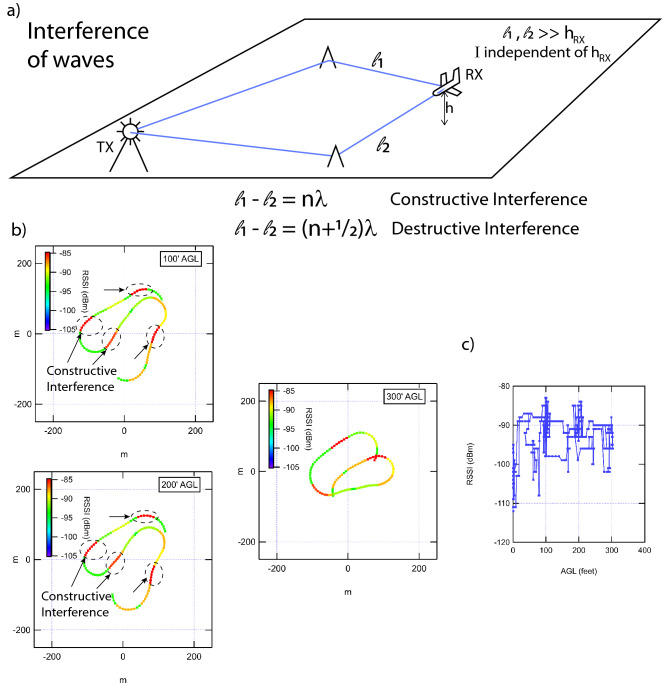
Top panel: Schematic of propagation from base station to airborne receiver (UAV). The blue lines are example rays the microwave may follow from base station the UAV. Bottom left panels: UAV flight path color coded by RSSI at three different altitudes (100′, 200′, 300′). The complex spatial pattern is almost identical at all three altitudes. Regions of high RSSI are circled in dotted lines, interpreted as regions where the waves interfere constructively. Right panel: RSSI vs. altitude for the entire flight, showing very little dependence on the altitude.

**Figure 5:**
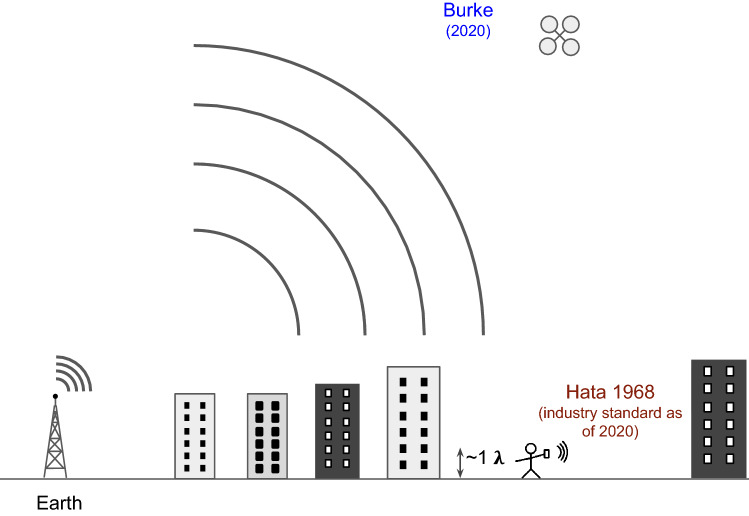
The Hata/COST321 model is the industry standard, but only covers heights near the earth, where scatting off of building, trees, etc. provides diffuse wave behavior. This work, in contrast, covers heights well above the ground, an unexplored territory.

**Figure 6:**
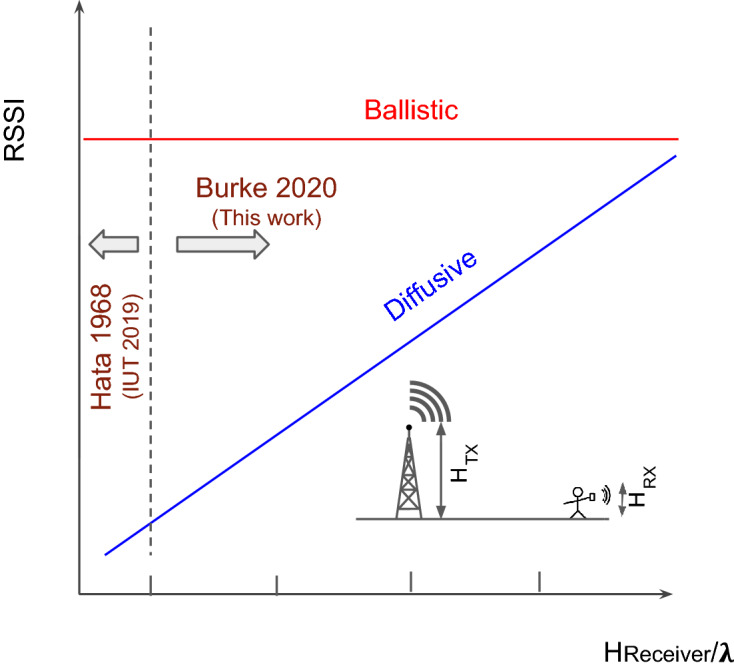
The Hata/COST321 model is the industry standard, but only covers heights near the earth, shown to the left of the dotted line. For ballistic, line of sight, the signal strength should be independent of height (assuming the height is much less than the distance to the transmitter), shown in red, as we observed in flight A. In contrast, for diffusive wave behavior, the higher the receiver, the less scatting matters, and the higher the signal strength (RSSI), as we observed in flight B. These are qualitative trends and must be quantified in future research.

### Flight C: wave interference from two strong scattering centers

In order to further investigate different regimes, we turn next to flight C, also in the diffusive regime, but in wide open relatively flat areas without mountains. In Fig. 4, the signal strength for the flight is shown in at three different altitudes (100′, 200′, 300′ AGL), as well as the signal strength vs. altitude for the entire flight (lower right panel).

This is perhaps the most interesting flight for several reasons. First, there is no overall position or altitude dependence of the signal strength. Again, this indicates this flight is in the diffusive limit, with multiple paths from the base station to the UAV contributing to the signal strength. However, what is perhaps most interesting is the strong (local) spatial dependence that persists from altitude to altitude and from pass to pass: There are specific regions of space where, as the UAV flies through, the RSSI increases by 10 dB or more, and then returns to the original value. This is clearly not Friis free-space loss dependence on position, other wise it would be monotonic. It is also unlikely to be purely diffusive propagation, as this would have a relatively homogeneous and bland (gentle) behavior on position.

The most plausible explanation of the strong spatial dependence is an interference phenomenon, due to only a few paths from base station to the UAV. This clearly indicates a new regime, that is somewhere in between ballistic and diffusive transport from base station to the UAV. A schematic explanation of the origin of the interference pattern is shown in the upper panel of Fig. 4.

This wave interference pattern has been observed before in the radio range: In the 1960s, two radio antennas were placed on two different high rise buildings in downtown Chicago in order to increase signal strength to the citizens of Chicago. This had the inadvertent effect of causing interference fringes and the radio station signal demonstrated hi/low behavior as one went from house to house! In our work, we are seeing similar behavior in the microwave regime.

## Discussion

Although propagation at high altitudes have been studied for aircraft purposes, this is the first ever comprehensive UAV data set of propagation at altitude under 400′ (see prior art discussion below). Propagation modelling for wireless is a vast field, but existing models are formulated to apply to at or near ground level receivers (Fig. 5). Therefore, it should be no surprise that existing models do not apply. Nonetheless, we can discuss the deviation from existing models to elucidate some of the features we have found in wave propagation that should be included in more sophisticated models in the future.

### Flight A

The first flight clearly has some distance to transmitter dependence that is not Friis like, i.e. there is some scatter. Existing models treat scattering off the earth and building as a separate path from transmitter (TX, base station) to RX (airborne receiver). If the Earth is treated as a perfect mirror, it is called the 2-ray or “plane earth” model. (See fig. 2.6 of Ref.^12^.) If the Earth is treated as a rough scatterer, there is the Sarkar model (based on Sommerfeld 100 years ago)^10^ and empirical models called the Hata-COST231 model^2,3^. We discuss how they compare to our data below.

### Comparison to ballistic (scatter free) propagation model

The predictions of a ballistic (scatter free) model would be the following:

- Altitude independence
- Path loss goes as 20 log (d) (Friis).

This assumes the altitude is much less than the distance to the transmitter, so that the horizontal distance d is approximately equal to the total distance from TX to RX (see Fig. 2). In our data, we observe altitude independence, which indicates minimal scatter. However, the path loss dependence on distance is not 20 log d, which we discuss next.

The location of the call tower is not known. Therefore the distance is not known, and the power law is not known. By adjusting the distance, a variety of power laws can be fitted, from 20 to 50 dB/decade of distance, but these are not quantitative since the distance is unknown with enough precision.Therefore, the actual power law is not really known. That being said, there IS a strong distance dependence, and NO altitude dependence in this case, which is opposite the mountainous case (flight B).

### Comparison to the 2 ray model

The 2-ray model assumes ballistic (scatter free) propagation from TX to RX along one path, and a perfect specular reflection from the ground (modeling the Earth as a perfect conductor i.e. mirror) as a second path, and does not take into account any other scattering or multi path, e.g. no diffusion or diffraction is modeled in the 2-ray model (see fig. 2.6 of ref.^12^).

The predictions of the 2-ray model are given by:

2 $$\begin{aligned} {P_r\over P_t} = G_{TX}G_{RX}\left( {h_{TX}h_{RX}\over d^2}\right) ^2 \end{aligned}$$

where $h_{TX}$, $h_{RX}$ are the heights of the transmit and receive antennas, respectively, and G the gains of the antennas. The assumptions of the 2-ray model are that the height of the receiver $h_{RX}$ and transmitter $h_{TX}$ are much lower that the distance d. Since the distance is over 1 km and the max height is 100 m this is reasonable. The interesting point about the data for flight A is that the RSSI is independent of the altitude over a large range of altitudes, from 100 to 400 ft. (30–120 m) (100–400 lambda). This is clearly not what is predicted by the 2-ray model, which predicts an $h_{RX}^2$ dependence on signal strength.

### Comparison to the Sarkar model

The Sarkar model^10^ predicts a 30 dB/decade decay (but only in the far-field, see below), for altitudes close to the ground. Our data shows a power law decay so is consistent in this sense. There is no comprehensive, analytical prediction for higher altitudes in this model in the far field. However, recent simulation work has addressed the near field, which we discuss in more detail below.

### Comparison to the Hata-COST231 model

The Hata-COST231 model is purely an empirical model. Through many iterations and refinements, it has been codified in a literal industry standard as ITU 2019^3^. However, the model, being empirical, has limitations, which are of course based on the input data range in the first place! In their case, they did not have drone technology. The model limits $h_{RX}$ to 1 to 10 $\lambda$. We are at well over 100 $\lambda$. Nonetheless, it is interesting to extrapolate, discuss, and compare.

Hata initial observed a “height gain” with antenna height, but only data up to 10 m in $h_{RX}$ was considered. However, there is no empirical prediction above this altitude. (We recently studied this in detail at low altitude, in a sister paper^13^). The general concept is that, as the receiver is raised, scattering becomes less prevalent. Extrapolation of Hata would give a huge dependence on altitude, which we clearly do not see. Therefore, Hata model does not really apply to flight A, nor does it give predictions even if extrapolated to our case. A similar conclusion can be drawn about the most recent industry standard COST231 which is based also on Hata’s work^3^.

### Near vs. far field and “breakpoint”

In all of the scatting models above, the predictions implicitly assume that the radiation is in the far field. The far field is traditionally assumed to begin at distances larger than $2l^2/\lambda$, where *l* is the maximum antenna dimension. However, in the context of scattering off the earth, the situation is not quite so straightforward. Sarkar has shown^14–16^ that for distances closer to the transmitter than $R_{breakpoint}$, the fields are actually near field:

3 $$\begin{aligned} R_{breakpoint}=4 h_{TX} h_{RX}/\lambda \end{aligned}$$

A common feature of all models is that they predict power law like behavior for the distance dependence for distances larger than a “breakpoint”, where the fields are already in the far-field region.

For distances *closer* to the transmitter than the breakpoint, since the fields are in the near-field region, there are no analytical predictions for propagation loss, per se. The reason is that, in the near field, the TX and RX coupling depends on more than just the distance and polar angle between the two. Near-field effects are not possible to predict analytically for general cases, and depend strongly on specific geometries. The exact definition of far-field vs. near field has been extensively discussed in^17^.

For most uses cases, where the receiver is near the ground, the breakpoint is a few hundred meters, meaning propagation studies and actual deployed systems will be beyond the breakpoint within the cell for a given tower. The case of drones is very different, where the altitude is high. For example, in our measurements here, the breakpoint is roughly 10 km assuming $h_{TX}=1 0$ m, since our drone flies up to 100 m. Therefore, our receiver (and by extension the use case of drones in general) is closer to the cell tower than this breakpoint. What that means is that *all* of the propagation models fail to be applicable to our data, and for drones in general. *There is no propagation model for drone-to-ground signal propagation.*

The implications of this are that our data (and drones in general) are not covered by the traditional models and comparison must be made to near-field simulations. We turn next to comparison with existing near-field simulations.

### Comparison to near-field simulations

In the near field, Sarkar has simulated the path loss for the 2-ray model as well as his own model, for a few different specific conditions. What is characteristic of the specific cases in the near field covered (i.e. simulated^14–16^ and recently measured^18^) so far are two characteristics:

- Drastic oscillations of signal strength with distance
- Very strong, counter-intuitive dependence on transmitter height (e.g. higher transmit gives lower signal strength)^19^.

High altitude receivers were not studied, but in one simulation study^14–16^, a 500 m transmitter gave actually *lower* signal strength than a 100 or 10 m transmitter at distances closer than the breakpoint.

Again, although we are in the near field according to this analysis, we do not see strong oscillations in signal strength with distance and we do not see strong altitude dependence. This also contradicts the (very few) simulations in the published literature on near-field propagation models.

### Summary flight A

Our flight A shows some very clear signs that scattering is minimal and that the characteristics are closest to that of ballistic nature. A detailed consideration of all of the existing models (including physics based models as well as purely empirical models) fail to give predictions for this altitude regime because they are all formulated for ground level receivers. In specific cases where trends can be predicted based existing models, the data we measure fails to obey these trends. Thus, not all is solved. There remain some enigmas which are not currently understood.

Therefore, this data we have presented here is significant and new. At this point we can only summarize the qualitative features of the propagation that will be used for future more comprehensive models. In addition, we point out that the data is new and significant, so is the method. In fact, our invention of this method should enable new models for the drone-to-ground propagation case in the future. Any new models will need to be checked and our method is a new, novel, and the best way to do that.

### Flight B

Flight B shows a very strong dependence on altitude, in contrast to flight A.

### Power law

We are unable to extract a meaningful power law. For the 100′, 200′ data there is clearly a change of about 25 dB over 500 m (200′) or 20 dB over 500 m (100′), but the high altitude date interestingly does not show any clear distance dependence. The 300′ is a band switch event as per our interpretation, where the carrier temporarily used a different band. We have since improved our software (http://www.gitlab.com/pjbca/4GUAV) to record the band at each flight but it was not recorded on this flight.

### Comparison to Hata, Sarka, 2 ray model

All three models predict an altitude dependence, which is consistent with our data, and this is also consistent with our interpretation that the flight is in the diffusive regime. However, none of those models take mountainous terrain into account, therefore our interpretation is an extension of the concepts of diffuse , multi path scattering to mountainous terrain. The 10 dB gain per 100′ of altitude (100 lambda) is empirical and we await more quantitative modeling based on terrain to explain our data.

### Flight C

Flight B shows little dependence on altitude OR position, in contrast to flight A. However, it shows strong wave like (interference) properties. This seems to be most consistent with the near field characteristics discussed above.

### Single scattering vs. diffusion

The two extremes of wave propagation regimes [ballistic and diffuse (Fig. 1)] are illustrated through two different sets of experimental data (flights A, B) in this work. However, the actual experiments are likely a combination of both. The main conclusion of this paper is that both can contribute to the propagation characteristics and both regimes can be realized in the physical world, not just in abstract mathematical manipulations, and that the regime that dominates depends on the local scattering environment.

There is one more in-between case this is important to consider, which is the result of interference of the direct wave with those scattered at most once (or a small number of times) off of a smooth ground. This is indeed one of the most well-known models in wireless signal propagation theory, and is called the “two ray” model or the “plane earth” model. In the two-ray model, the direct path (ray) from transmitter to receiver interferes with the ray reflected specularly off the surface of the earth, which is model as a perfect conductor. The earth is assumed to be smooth in this model^12^. In this model, the propagation loss is predicted to be 40 dB/decade, which is not what we observe. Therefore, the experimental data we present is not consistent with a model which is the result of interference of the direct wave with those scattered at most once (or a small number of times). For this reason, we find the diffusive interpretation more appropriate. An extensive discussion of scattering off of multiple sites with many lines (or rays) from transmitter to receiver has been developed by Sarkar^10^. It therefore seems that the description as diffusive is the most appropriate interpretation of the experimental data, although at present there is no diffusion based model that can predict or even postdict our experimental data. We would like to again emphasize that this work is primarily a data driven exercise. While the data contradict existing models, there is no model at present that explains all the data we present herein. In our opinion, models are fine as exercises in thought. However, experimental data is what makes up the real world, so models must be subservient to data, and not the other way around. This is especially true in engineering fields such as wireless communications, where system performance and economic impact are ultimately beholden to real system performance data and physical reality, not mathematical models thereof.

### Scattering vs. absorption

In addition to scattering, electromagnetic fields also experience absorption. However, one must be careful when one uses the word absorption. One meaning is the complete loss of the signal upon encountering an obstacle. However, this assumes the obstacle blocking the path reflects the wave and the wave never makes it to the receiver. This is in fact not what is assumed in the Hata-COST321 and subsequent models. Those models explicitly allow for propagation from transmitter to the receiver even if there is no direct line of site. In that case, there is propagation but only after scattering off of many scattering sites (buildings, trees, etc). So this definition of absorption is equivalent to the Hata-COST321 model and its subsequent refinements. The other definition of absorption is where energy is lost due to resistive currents in scattering centers, like a black body absorbing rather then reflecting light. (We recently demonstrated this regime by perfectly impedance matching a material to free space to get of order unity absorption of RF^20^, but this is not normally the case in the physical world we live in.) Because this is not typically the case in the RF regime, it is unlikely to be a significant contributor to signal loss, and has not been modeled in the industry standard Hata-COST321 and refinement models, and is not considered as a significant effect here.

### Summary of discussion

None of the existing models were developed for drone-to-ground communications, and therefore this data (and method) is a fundamentally new area of research that we are opening up. We have shown this through detailed analysis of each model, and each flight.

Although the models are not developed yet, we have shown three significant aspects that appear in physical reality: Ballistic wave propagation, diffusive wave propagation, and strong interference of only a few paths. This lays the experimental and intellectual foundation for the field of drone-to-ground propagation models and our method can be used to verify it. Drone-to-drone communication is also new frontier that this work enables.

## Comparison to prior art

### Data from UAVs

There have been previous attempts to use UAVs to determine signal coverage. We reviewed all of these in^11^. Briefly, the only other published work which comes close to the depth and breadth of the work presented in this paper is the 2017 study by Qualcomm^21^. That study, while comprehensive in its data set, focused on switching from tower to tower, as well as re connection delay, rather than wave propagation loss which we have emphasized in this paper. The tower to tower switching time and re connection time is really a function of the detailed protocol used by the carriers, and this information, as well as the tower being used before and after a switch, is not generally accessible through public APIs and may be considered proprietary by some carriers. Therefore, while the Qualcomm study demonstrated the power of UAVs, it focused narrowly on the cell to cell switching and lag (latency), something that is quiet carrier specific. In contrast, our study here has demonstrated a more ubiquitous data set and focused on wave propagation, a more universal phenomenon of general significance to all wireless communication in the GHz, mm-wave, and even THz bands.

### Models in 3D vs. 2D

Our work is not a path loss measurement but rather a measurement “in the wild” of ACTUAL deployed cellular systems. We would also like to point out that, although site-specific predictions of signal strength both indoor and outdoor based on detailed terrain and obstruction (buildings, floor plans, etc) data is a research area for electromagnetics in wireless communications, that our technology enables for the first time economical surveys of the resultant 3D signal distribution, something that was previously not possible.

The diffusive/ballistic interpretation is just a first stab at the data interpretation. Just as Hata did in^2^ based on detailed ground based measurements performed in 1968 by Okumurato^1^, we anticipate new models will be developed based on this measurements presented here in. It should be noted that the height dependence we present here is completely not predicted in existing models. This is a new territory. A proposed description is in Fig. 6.

## Perspective and future applications

Engineers like formulaic predictions for all use cases. However, with electromagnetic waves, the scatting sites cannot be predicted. Perhaps it would be a better approach to have a site-specific propagation model, rather than trying to come up with a universal model that covers all cases. Until our work here-in, that has not been possible because of the prohibitively expensive nature of signal mapping at ground level. Drones have the potential to lower this cost and provide site specific, low cost, 3D signal propagation maps at every site in the world. What can one do with that information?

Detailed knowledge of signal propagation can give rise to dramatic and revolutionary exploitation of waves. For example, in a swimming pool, reconstruction of the wave front details can allow a reverse wave to be created, causing a pebble drop outward propagation wave to be time reversed computationally and in the physical world^22^ (https://www.youtube.com/watch?v=XDbLi2YGQn8). Optical wave propagation in random media, long thought to be impossible to measure and manipulate experimentally, is now possible to measure, compute, and exploit^23–29^. With the ubiquitous nature of wireless communications connecting every human on the planet, it may be possible to similarly exploit detailed experimental (big data) sets to enable as yet to be invented methods of exploitation of waves in the GHz to mm wave range for interconnecting humans and “things”. The work presented in this paper is a first and significant step in this direction, and would be an enabling technology for such signal propagation exploitation in the GHz to mm-wave (and possibly even THz) range. Similarly, just like Google car has street view of the entire world by driving cars around, one could fly a wing around and have a cell phone coverage map of the entire planet.

We would like to elaborate on this concept. Typically, in the wireless world, the implicit assumption has been, that if the signal strength is low, the information capacity is diminished, because the signal to noise is diminished. We would like to challenge that assumption and this work provide an experimental method to do demonstrate new avenues of exploitation of channel capacities where the path loss is not the only or even dominant figure of merit. The idea that information can be transmitted through disordered media by diffuse waves has been discussed for some time in the literature from a theoretical perspective^30,31^, but now there is finally the possibility to exploit these concepts in actual hardware implementation at scale. Thus, in addition to new models of path loss, this work opens new methods for information and communication.

Note that this technology could be used for more than just RSSI mapping. It could be used for anything that can be sensed on a UAV, e.g. atmospheric conditions (pressure, temperature, humidity); environmental conditions (e.g. pollutants and toxins); traces gasses; even microorganisms (bacteria), as long as suitable sensors can be developed.

## Conclusion

The Hata-COST321 model has served the wireless communication industry well for over 50 years for ground level receivers. Drones are a new era, and currently there is not Hata-COST321 equivalent “model” that covers the propagation. Such a model is imperative to develop if widespread drone deployment is to become a reality. The FAA is even proposing to require an internet connection for some or all drones, which would lead to millions of internet connected drones, making the requirement of a realistic propagation model imperative for the future safety and economy of all aviation. No matter what that propagation model ends up being, it will have to have the ballistic nature (flight A) and diffusive nature (flight B) and wave interference nature (flight C) included in it, which we have presented in this work based on our first in class propagation measurements with drone technology. At the same time, drones can go where no man can go and map fields in a way never possible in the past, which may open many new applications for manipulation and exploitation of the unique properties of electromagnetic waves.

## Methods

### Vehicle description

Details of the vehicle and the methods are presented in another paper^11^. Briefly, the system is a 600 mm flying wing with an STM32 F4 based bit flight controller running Ardupilot with GPS and autopilot capabilities capable of complete autonomous flight, from takeoff waypoints to landings. An on-board Linux companion single board computer (Raspberry Pi Zero W) handles communication with the 4G modem and internet. The UAV can be piloted remotely via a separate 900 MHz transmitter or via the 4G internet connection over the internet.

### 4G internet connection

A Novatel 4G modem (model # USB 720L) interfaces with the companion computer and provides an IP connection to a cloud based Linux instance running on Amazon Web Services, which then connects to a Windows 10 computer on the ground. (The code is open source and available in a git repository at http://www.gitlab.com/pjbca/4gUAV). The modem antenna is a 2 dB dipole antenna with vertical polarization, which protrudes above the craft (Fig. 1) so as to minimize interference from any other components.

### RSSI recording

The Novatel firmware provides an html server to the on board computer which provides an RSSI in dBm. The RSSI is recorded continuously to a local SD card on the Linux on board companion computer, and the time stamp is used together with the log files to coordinate the craft location in 3D with less than 1 m error bar. Post flight, the RSSI and GPS coordinates are matched in software to provide a 3D coverage map.
